# The Postembryonic Development of the Immunological Barrier in the Chicken Spleens

**DOI:** 10.1155/2019/6279360

**Published:** 2019-05-05

**Authors:** Qian Zhang, Xuejing Sun, Taozhi Wang, Bing Chen, Yufei Huang, Hong Chen, Qiusheng Chen

**Affiliations:** ^1^Key Laboratory of Antibody Techniques of National Health Commission, Nanjing Medical University, Nanjing 211166, China; ^2^MOE Joint International Research Laboratory of Animal Health and Food Safety, College of Veterinary Medicine, Nanjing Agricultural University, Nanjing 210095, China

## Abstract

The avian immune system improves with the development of the lymphoid organs. The chickens' spleen serves as the largest peripheral lymphoid organ, but little immunological research has been conducted on that spleen during postembryonic development. We investigated the blood-spleen barrier (BSB) by developing morphological architecture, resistance to the corpuscular antigen, immunocyte distribution, gene expression levels of TLR2/4 and cytokines in the spleens of hatched chickens of differing ages. Results demonstrated that the resistance of exogenous carbon particles of the BSB improved with the morphological and structural development of the chicken spleens. The cuboidal endothelial cells which lined the sheathed capillaries were gradually visible, and the discontinuous basement membrane was thickened during postembryonic development. There was an increased number of T and B cells and antigen-presenting cells in the chicken spleen between hatching and adulthood. The mRNA expression levels of TLR2/4, IL-2, IFN-*γ*, and TNF-*α* were higher two weeks after hatching, but these decreased and remain stable between 21 and 60 days. As the age increased, the BSB developed structurally and functionally. Our findings provide a better understanding of splenic immune function and the pathogenesis of avian immunology in infectious diseases.

## 1. Introduction

The spleen is the largest peripheral lymphoid organ in chickens, and it plays a significant role in both antibacterial and antiviral immune responses against acquired antigens. Lymphocytes which migrate to the avian peripheral lymphoid organs are derived from central immune organs which proliferate and differentiate during embryonic development. Development of the peripheral lymphoid organs is closely related to immune function maintenance [1]. During embryonic development, the spleen functions as a hematopoietic organ. Following the migration of lymphocytes and the formation of red and white pulps, the spleen plays a key role in immune responses, particularly to blood-borne antigens [2]. Embryonic mammals are granted access to the innate immune system of the maternal antibody via the placenta, while in egg-laying birds, the immune mechanism differs. It has been reported that maternal immunoglobulins from the hen are first isolated in the yolk of maturing oocytes before being transported across the yolk sac to the circulation of the developing chicken [3, 4].

Since the chicken's immune system lacks lymph nodes but has the bursa of Fabricius, it has a crucial position in phylogeny. There are substantial structural and functional differences between the immune systems of birds and those of mammals. These differences include the major histocompatibility complex (MHC) and the somatic recombination in the generation of antibody diversity [5, 6]. There are an increasing number of studies exploring immunological responses in the chicken spleen [7, 8]. As a consequence of the poorly developed lymphatic vessels in avian species, the chicken spleen parenchyma is divided into two differentiated compartments—red and white pulps—without evidence of a marginal zone. The blood-spleen barrier (BSB) has been characterized as a filtration bed and is located in the splenic marginal zone of rodents [9, 10]. Previous research has identified the BSB in chickens, describing a reticular framework presented in the splenic ellipsoid and PELS which protects the spleen from the invasion of circulating pathogens [11]. There is limited information about the morphological features and immunological mechanisms of the BSB in mammals, as well as on the structure and function of BSB in chickens during postembryonic development.

The innate immune system is a crucial contributor to the inflammation induced by microbial infection or tissue damage. Toll-like receptors (TLRs) are important pattern recognition receptors (PRRs) in the innate immune system, which are expressed on both immunocyte and nonimmune cells, meaning it is the first barrier formed by the innate immunological defense [12, 13]. TLRs operate as sentinels of the innate immune system. Recognition of pathogen-associated molecular patterns (PAMPs) by TLRs results in the inflammatory immune response being characterized by the production of reactive oxygen, nitrogen intermediates, and proinflammatory cytokines. Chicken TLRs consist of 10 genes which resemble those of humans and two fewer than found in mice [14]. Different TLRs play crucial roles in the activation of the immune response. Higgs et al. reported the transcriptional level of TLRs both during embryonic development and following Salmonella enterica serovar typhimurium infection, which found that Salmonella enterica infection resulted in a significant induction of TLR4. TLR2 and TLR4 mediate an oxidative burst in chicken heterophils when they are stimulated with lipoteichoic acid and LPS [15]. However, it remains unclear whether TLR2/4 expression in the chicken spleen after hatching is associated with the development of the BSB of the innate immune system or if there is any difference in the expression of TLR2/4 at different ages of the development of the chicken spleen. The production of cytokines involved in the early stage in immune response mediates the effects of innate and adaptive immunity. Cytokines are produced by different types of cells and are able to exert regulatory effects on both hematopoietic cells and immunocytes which are involved in host defense and homeostasis [16]. However, cytokines utilized in the development of immunocyte have not been well described in the immunological barrier of the chicken spleen. In order to further understand the immune function during the postembryonic development of the chicken spleen, we explored the immune barrier and the development of the morphological architecture, resistance to the exogenous carbon particles, immunocyte distribution, and immunological gene expressions in the chicken spleens of differing ages after hatching. A better understanding of the immune function of the BSB during the postembryonic development of the chicken spleen has the potential to improve our understanding of avian immunology and pathogenesis of infectious diseases in chickens.

## 2. Materials and Methods

### 2.1. Animals

All experiments were conducted according to the Animal Research Institute Committee guidelines of Nanjing Agriculture University, China. SPF Sanhuang broiler eggs were incubated at 38.3°C, 70% relative humidity for two to three days. The hatched chickens were maintained in a specific pathogen-free condition and were not vaccinated against any diseases. Sanhuang broiler chickens of 1, 7, 14, 21, 35, and 60 days old were used in this study. All animals were euthanized by cervical dislocation after intravenous administration of 3% sodium pentobarbital (25 mg/kg). The slaughtering and sampling procedures were approved by the Nanjing Agricultural Veterinary College Experimental Animal ethics committee; the approval ID is SYXK (SU) 2010-0005.

### 2.2. Ink Injection

Chickens of different ages were injected with Indian ink (5 ml/kg, Aolindan) containing 50-100 nm of carbon particles. India ink diluted 1 : 10 with PBS was injected intravenously after anesthesia with 3% sodium pentobarbital (25 mg/kg, Sigma-Aldrich). Chickens in the control group were intravenously injected with the same volume of PBS. There were five chickens in each group. The spleens were collected 30 min after injection, and formalin-fixed paraffin sections (5 *μ*m) were assessed using light microscopy with or without HE staining.

### 2.3. Transmission Electron Microscopy

Spleen samples were obtained immediately after postmortem. Samples were cut into small blocks (1 mm^3^), immersed in 2.5% glutaraldehyde fixative in 0.01 M phosphate-buffered saline (PBS; pH 7.4) at 4°C overnight. They were then submerged in 1% osmium tetroxide in the same buffer for 60 min. Samples were dehydrated in ascending concentrations of ethyl alcohol being before infiltrated with a propylene oxide-Araldite mixture and embedded in Araldite. Ultrathin sections were stained with uranyl acetate and lead citrate for 20 min each. Sections were examined and photographed using a transmission electron microscope H-7650 (Hitachi).

### 2.4. Immunohistochemistry

Spleen samples were fixed in 4% buffered paraformaldehyde for more than 24 h and were embedded in paraffin. Paraffin sections (5 *μ*m) were cut and stained according to immunohistochemical methods [17]. Sections were inactivated with 3% hydrogen peroxide for 10 min and then blocked with 5% bovine serum albumin. The primary anti-chicken antibodies CD3 (CT-3, 8200-01, Southern Biotech), Bu-1 (AV20, 8395-01, Southern Biotech), and MHC II (21-1A6, ab34031, Abcam) were incubated at 4°C overnight at a 1 : 150 dilution. Biotinylated goat anti-mouse IgG (Boster Biotechnology) was used as the secondary antibody. Avidin-biotinylated peroxidase complex and DAB (Boster Biotechnology) were utilized for development. Negative control with PBS was used instead of primary antibodies. Images were captured using an Olympus microscope (BX53).

Cell count measurements were performed using Image-Pro Plus 6.0 (Media Cybernetics, Silver Spring, MD). Positive areas were expressed as the integral optical density (IOD). Mean results of six visual field per section were used to calculate IOD.

### 2.5. RNA Isolation and qPCR Analysis

Total RNA was extracted from the chicken spleens using the TRIzol reagent (Invitrogen, Carlsbad, CA, USA) according to the manufacturer's instructions. Extracted RNA was then reverse transcribed into cDNA using the SuperScript First-Strand Synthesis System (Invitrogen, Carlsbad, CA, USA). Quantitative real-time PCR (qPCR) was performed with the MyiQ2 Real-Time PCR System (Bio-Rad, California, USA). The PCR system was performed containing SYBR Green Supermix (Invitrogen, Carlsbad, CA, USA), 10 mM specific primer, and 0.1 mg of the template cDNA. Reactions were carried out with a denaturation step of 95°C for 10 s, followed by 35 cycles of 95°C for 5 s, 60°C for 30 s, and 72°C for 10 s. The relative expression of the target genes was referred to *β*-actin and then analyzed using the 2^-ΔΔCT^ method [18]. Each experiment was performed for three replicates. According to gene sequences in the NCBI database, specific primers were designed by the Beacon Designer 7.0 software (Premier Biosoft International, USA). Primer sequences used in this study can be seen in Table 1.

### 2.6. Statistical Analysis

All data were expressed as means ± SEM. Statistical analyses were performed using SPSS software version 14.0. One-way ANOVA followed by Duncan's test were used. *P* values of <0.05 were considered as significant differences.

## 3. Results

### 3.1. The Location of the Blood-Spleen Barrier after Embryo Development

**Figure 1:**
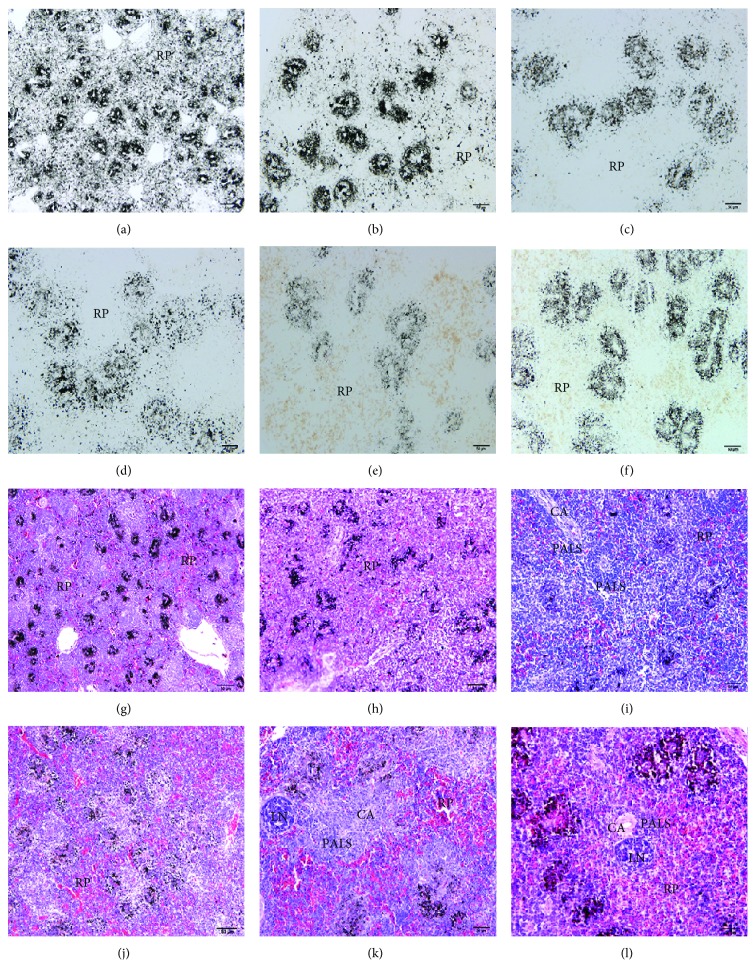
The BSB against the carbon particle in different ages of the chicken spleens with HE staining. (a, g) The histological structure of the chicken spleens at 1 d after hatching. (b, h) The histological structure of the chicken spleens at 7 d. (c, i) The histological structure of the chicken spleens at 14 d. (d, j) The histological structure of the chicken spleens at 21 d. (e, k) The histological structure of the chicken spleens at 35 d. (f, l) The histological structure of the chicken spleens at 60 d. The first two lines are paraffin sections dewaxing without HE staining. CA: central artery; LN: lymph nodule; RP: red pulp; PALS: periarteriolar lymphatic sheath.

In order to investigate the development of the blood-spleen barrier in hatched chickens, birds of differing ages were intravenously injected with India ink (Figure 1). Thirty minutes after ink injection, uptake of carbon particles was partly limited to the ellipsoid, with the most carbon particles dispersed in the red pulp one day after hatching. Before 14 days, there were also carbon particles in the red pulp. However, carbon particles in the red pulp gradually decreased after 14 days. There was almost no carbon in the red pulp, lymph nodule, and PALS in the spleens of chickens that were 35 and 60 days old. This suggested that the ability of ellipsoid-restricting carbon particles in the white pulp had increased.

### 3.2. Morphological Characteristic of the Chicken Spleen during Postnatal Development

**Figure 2:**
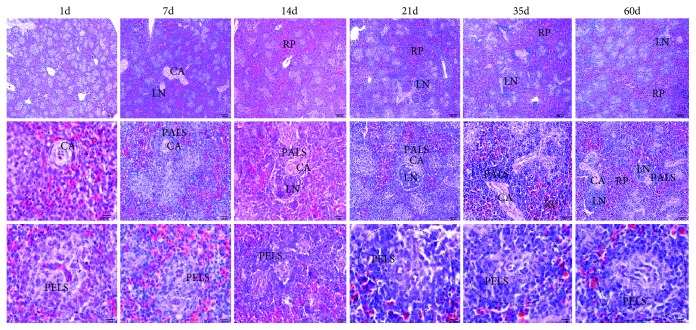
The histological structure of different ages of the chicken spleen with HE staining. Each column represents the histological structure of the chicken spleens of the same age. CA: central artery; LN: lymph nodule; RP: red pulp; PELS: periellipsoidal lymphatic sheath; PALS: periarteriolar lymphatic sheath.

One day after hatching, the chicken spleens consisted of white and red pulps (Figure 2). PELS developed earlier than PALS, and there were limited lymphocytes around the ellipsoid and PELS. Almost no lymph nodule or PALS was found one day after hatching. Instead, lymph nodule and PALS formed at 7 days. The area of PALS and lymph nodule increased and the structure of lymph nodule became clearly visible at 14 days. After 21 days, both PELS and PALS increased, and the structure of the chicken spleen tended to stabilize. At 60 days, the structure of the chicken spleen had developed completely. The diameter of PELS and PALS tended to increase and develop as showed in Table 2.

### 3.3. The Ultrastructural Endothelium of Splenic Sheath Capillary in the Chicken Spleens of Different Ages

**Figure 3:**
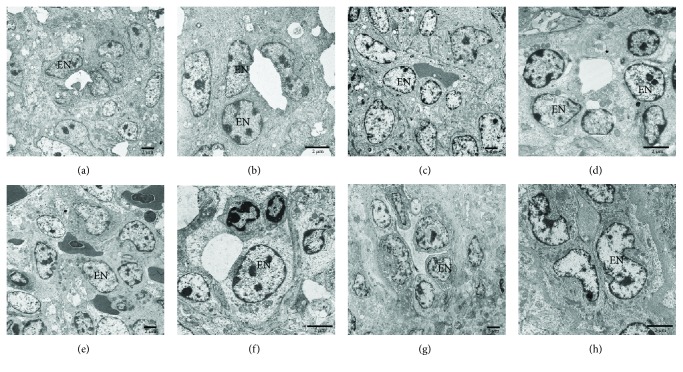
The ultrastructure of endothelial cells of the sheathed capillary in the chicken spleens of different ages. (a, b) The endothelial cells in a 1-day-old chicken spleen. (c, d) The endothelial cells in a 7-day-old chicken spleen. (e, f) The endothelial cells in a 35-day-old chicken spleen. (g, h) The endothelial cells in a 60-day-old chicken spleen. EN: endothelial cell.

Electron microscopy observation demonstrated that the endothelial cells of the sheathed capillary were sparse; the intercellular space was comparatively large; the lumen of the endothelial cells was small; and the basement membrane could not be seen clearly. At 7 days, the number of endothelial cells around the capillaries increased; the intercellular space became smaller and more compact; the endothelial cells displayed a clearly cubic shape; and the basement membrane was thinner than that of chickens at 60 days. At 35 and 60 days, the cuboidal endothelial cells were clearly visible, and the discontinuous basement membrane thickened as age increased (Figure 3).

### 3.4. Distribution of Lymphocytes and Antigen-Presenting Cells in the Blood-Spleen Barrier of Chickens

**Figure 4:**
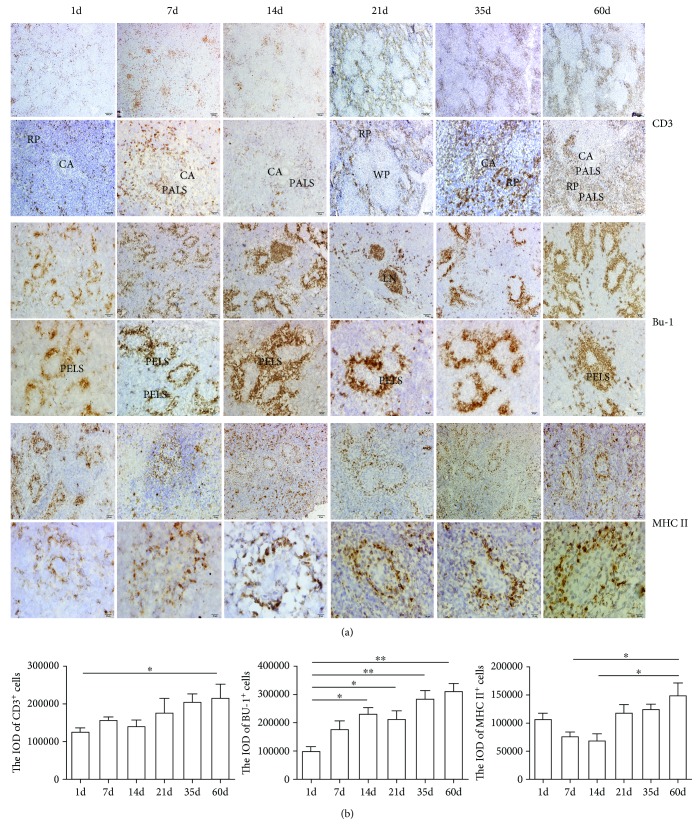
CD3^+^ T cells, Bu-1^+^ B cells, and MHC II^+^ cells in the chicken spleens of different ages. (a) The immunohistochemistry image shows the distribution of CD3^+^ T cells, Bu-1^+^ B cells, and MHC II^+^ cells. CA: central artery; RP: red pulp; WP: white pulp; LN: lymph nodule; PALS: periarterial lymphatic sheath; PELS: periellipsoidal lymphatic sheath. (b) The IOD of CD3^+^ T cells, Bu-1^+^ B cells, and MHC II^+^ cells in the chicken spleens of different ages. ^∗^*P* < 0.05, ^∗∗^*P* < 0.01. The results are representative of three independent experiments.

CD3 and Bu-1 are markers of T and B cells, respectively. Immunohistochemistry results showed T and B cells distribution in the chicken spleens of different ages (Figure 4). The CD3^+^ T cells of the chicken spleens were mainly distributed around the central artery and the red pulp. As the age increased, the distribution of the CD3^+^ T cells in the red pulp and around the central artery gradually increased. Bu-1^+^ B cells were mainly located in the ellipsoid and lymph nodules, although a few were located in the red pulp. Bu-1^+^ B cells displayed in a ring shape around the capillary one day after birth, while the area of the positive B cells around the ellipsoid increased from 1 to 60 days after hatching. MHC II is a class of major histocompatibility complex molecules, which is expressed on antigen-presenting cells including dendritic cells, phagocytes, and B cells. These cells are key for initiating immune responses. In our results, MHC II were positively expressed around the ellipsoid and red pulp, which are ellipsoid-associated cells and macrophages in the red pulp (Figure 4). MHC II-positive cells formed as the spleen developed. The distribution of MHC II-positive cells increased from 14 days up to adulthood, at 60 days.

### 3.5. The mRNA Expression of TLR2 and TLR4 in the Chicken Spleens of Different Ages

**Figure 5:**
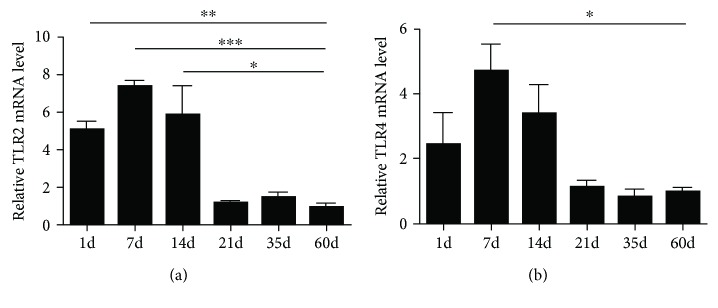
The mRNA expression levels of TLR2 and TLR4 in the chicken spleens of different ages. Values are the mean ± SEM. ^∗^*P* < 0.05, ^∗∗^*P* < 0.01, and ^∗∗∗^*P* < 0.001. Different ages of gene expression are compared with those of 60-day-old adult chickens.

In order to validate the role of TLR2 and TLR4 in the development of the chicken spleens, qPCR analysis has been used to detect levels of TLR2 and TLR4 mRNA expression in the chicken spleens of different ages (Figure 5). Results showed that TLR expression varied at different ages after hatching. Expression levels of TLR2 and TLR4 increased relatively from 1 day to 14 days. After 14 days, the expression of TLR2 and TLR4 mRNA in the chicken spleens was significantly reduced (^∗^*P* < 0.05, ^∗∗^*P* < 0.01, and ^∗∗∗^*P* < 0.001). In contrast, at 21 to 60 days, there was no evident change to the mRNA levels of TLR2 and TLR4.

### 3.6. The mRNA Expression of IL-2, IFN-*γ*, and TNF-*α* during Chicken Spleen Development

**Figure 6:**
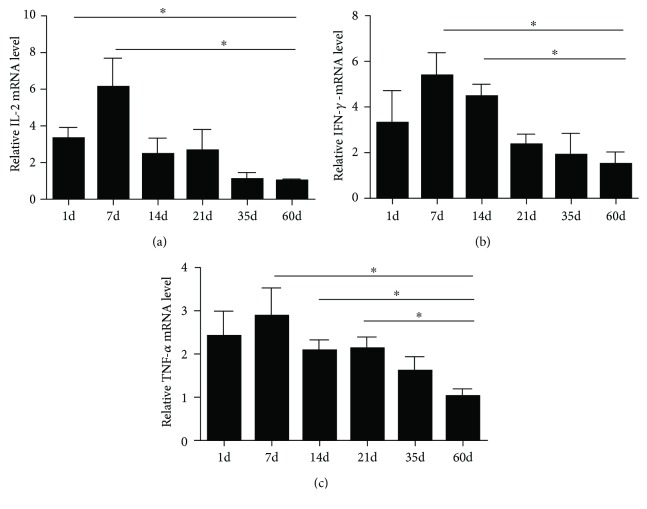
The mRNA expression levels of IL-2, IFN-*γ*, and TNF-*α* in the chicken spleens of different ages. Values are the mean ± SEM. ^∗^*P* < 0.05. Different ages of gene expression are compared with those of 60-day-old adult chickens.

In order to further investigate cytokine expression during spleen development, we detected IL-2, IFN-*γ*, and TNF-*α* mRNA expression in the chicken spleens of different ages using qPCR (Figure 6). Results demonstrated that, to a certain extent, cytokine expression varied regularly at different ages. Levels of IL-2 and TNF-*α* mRNA increased at 7 days, while lower levels were detected as age increased. The IFN-*γ* mRNA expression was higher at 1 day to 14 days, but lower at 21 to 60 days (*P* < 0.05). Taken together, IL-2, IFN-*γ*, and TNF-*α* mRNA were higher in the chicken spleens within two weeks after hatching and then gradually reduced until they stabilized in adulthood.

## 4. Discussion

There is limited research into the BSB in mammals, including the structure and function of the barrier of chickens during postembryonic development. Mast and Goddeeris have reported that the periarteriolar lymphoid sheath (PALS) and the splenic ellipsoids were beginning to form 20 days into the embryonic stage, while the periellipsoid lymphoid sheath (PELS) matured during the first week after hatching in broiler chicken spleens [3]. Zhang and Yang reported that PALS and PELS formed 4 days after hatching, and that the germinal center with distinct morphological structure was observed at 14 days [19]. In the previous study, the location and structure of BSB in chickens were reported [11], but the immune function and the manner in which the immunological barrier resists antigens during spleen development remained uncertain.

In this study, the resistance of the exogenous antigen of BSB with carbon antigen was investigated. Results showed the location of carbon particles in the chicken spleens of different ages. Carbon particles were partially limited around the ellipsoid, mostly dispersed in the red pulp, which suggested a weak physical barrier one day after hatching. As age increased, the resistance capability increased, which could be due to the morphological and structural development of the chicken spleen.

Regarding that morphological characteristic, PELS was found to develop first without the presence of splenic nodules or PALS. There were fewer lymphocytes around PELS and the area of the capillary sheath was limited. Electron microscopy demonstrated that the intracellular space of the adjacent endothelial cells was incompact; the lumen was narrow and the basement membrane which lined the endothelial cells was not clearly visible 7 days after hatching. The failure of the chicken spleen to resist the carbon particles at the initial stage was due to the incomplete development of the spleen architecture, particularly the ellipsoid structure and the sheath of the capillaries. As the chickens reached adulthood, the basement membrane thickened. The area of lymphoid tissue PELS and PALS gradually increased, while lymphocytes and antigen-presenting cells were distributed in the white pulp with the development of the spleen, suggesting the resistance ability of the BSB increased.

It has been reported that immunocyte and immune components have been found in the embryo at 21 days [20]. During embryo development, precursor T and B cells migrated from the thymus and bursa of Fabricius to the peripheral lymphoid organs—spleen and lymph node [21, 22]. As T and B cells develop in the lymphoid organ, the avian immune mechanism gradually improves. CD3 and Bu-1 are markers of T and B cells. CD3 and TCR molecules constitute the TCR complex, which induced the activation signal in T cells. Bu-1 expressed on chicken B cells throughout most of those cells' development [23, 24]. Immunohistochemistry results of CD3 and Bu-1 distribution reveal the development of T and B cells in the chicken spleen. The number of CD3^+^ T and Bu-1^+^ B cells in the chicken spleen increased from 1 day to 60 days, although this increase was not linear. The distribution of CD3^+^ T cells within the first 7 days after hatching was more than on the first day after birth. However, this showed a downtrend at 14 days. In contrast, the positive Bu-1 B cells at 21 days were less than those at 14 and 35 days. In general, the distribution of T and B cells increased between hatching and adulthood. MHC II is a class of major histocompatibility complex molecules which is expressed on antigen-presenting cells. The MHC II-positive cells expressed around the ellipsoid can first capture and then present antigens from the blood, providing a cellular basis for the innate immune function of BSB.

TLRs are expressed in tissue-specific nonimmune cells, including the alimentary tract, the respiratory tract, and the reproductive tract [25–27], suggesting it is the first barrier in innate immunological defense. There is increasing information available about chicken TLRs. Several studies have explored TLR ligands as a therapy to provide protective immunity against various infectious diseases in chickens [28–30]. However, such research has not described TLR expression in the chicken spleen after hatching in a typical condition. In order to investigate whether TLRs regulate the maintenance of the immune function of the BSB in chickens, we identified TLR2 and TLR4 gene expression levels in the chicken spleens of different ages during postembryonic development. Our results demonstrated that expressions of TLR2 and TLR4 mRNA were higher within the two weeks after hatching, but that they decreased and remained relatively stable from 21 to 60 days. While this tendency may be not in accordance with the development of the chicken spleens, these different expressions of TLR2 and TLR4 are closely related to the immune function of the chicken spleen.

The downstream of TLRs signaling pathways via the activation of transcription factors leads to the expression of several immune genes, including cytokines, chemokines, adhesion molecules, and receptors [31]. The production of cytokines plays a key role in the early stage of immune response, mediating the effects of innate and adaptive immunity. IFN-*γ*, TNF-*α*, and IL-2 are pleiotropic cytokines which are utilized during proinflammatory response. IFN-*γ* and IL-2 are T cell proliferation factors that are released from Th1 cells and are essential cytokines for expansion of activated T cells. TNF-*α* which has been produced by leucocytes and endothelial cells is involved in the infection caused by bacteria, viruses, and parasites. In addition, TNF-*α* also regulates the inflammatory response and apoptosis of tumor cells [14, 32]. These cytokines in the immune organ showed the immune function of the chicken spleens during development.

Then we investigated the mRNA expression levels of IL-2, IFN-*γ*, and TNF-*α* in the chicken spleens of different ages. We found that IL-2, IFN-*γ*, and TNF-*α* mRNA were higher within two weeks after hatching and then gradually reduced to stability until adulthood. Gu and Li reported that the gene expression of IFN-*γ* coincides with the chicken spleen development and the migration of lymphocytes [33]. However, our immunohistochemistry results showed that the CD3 and Bu-1-positive cells increased as age increased, which does not appear to be in accordance with the cytokine expression during development. When combined with the architecture and immune function of the chicken spleen, it seems reasonable to speculate that the barrier function was immature right after hatching, and that this leads to the increased level of the cellular immunity, while the activation of T and B cells migrating to the peripheral lymphoid organs from the embryo to be hatched may be due to the rise of cytokines immediately after hatching. As the chicken spleen developed, the barrier function improved, and lymphocytes, macrophages, and antigen-presenting cells around the splenic ellipsoid became capable of capturing antigens instantaneously. Taken together, the histological characteristics of the chicken spleen, especially the architecture of the BSB, provide a structural basis for the immune function which resists the exogenous antigen. As the important peripheral immune organ, the spleen is inclined to enhance immunological levels by regulating the activity of immunocyte and the immune defence. The strict maintenance and regulation of the splenic immune function in chickens is an important feature of avian immunology. Further investigation into the ontogeny and function of the immunological barrier could make a substantial contribution to our understanding of the mechanisms of the avian immunology as well as providing a better means for studying the role of the chicken spleen in inflammation and immunological reactions in infectious diseases.

## 5. Conclusion

Our results suggest that BSB developed with the morphological architecture, which is resistant to the corpuscular antigen, immunocyte distribution, TLR2/4, and cytokine expressions in the postembryonic chicken spleen and which provides a structural basis for the splenic immune function as well as a better understanding of avian immunology and the pathogenesis of infectious diseases.

## Figures and Tables

**Table 1:** Primers and conditions used in the qPCR.

Target genes	GenBank accession	Primer sequences (5′-3′)
TLR2	XM015301380	F:ATCCTGCTGGAGCCCATTCAGAG
		R:TTGCTCTTCATCAGGAGGCCACTC
TLR4	NM001030693	F:AGTCTGAAATTGCTGAGCTCAAAT
		R:GCGACGTTAAGCCATGGAAG
IL-2	XM015276098	F:CAAGAGTCTTACGGGTCTAAATCAC
		R:GTTGGTCAGTTCATGGAGAAAATC
IFN-*γ*	NM205149.1	F:GACAAGTCAAAGCCGCACA
		R:TCAAGTCGTTCATCGGGAGC
TNF-*α*	XM015294125	F:GGACAGCCTATGCCAACAAG
		R:ACACGACAGCCAAGTCAACG
*β*-Actin	NM205518	F:GAGAAATTGTGCGTGACATCA
		R:CCTGAACCTCTCATTGCCA

**Table 2:** The diameter variation of PELS and PALS in different ages of the chicken spleen.

Age	Mean diameter of PELS (*μ*m)	Mean diameter of PALS (*μ*m)
1 d	11.92 ± 0.91^d^	5.67 ± 0.68^d^
7 d	17.97 ± 1.08^c^	22.82 ± 1.05^c^
14 d	24.27 ± 1.29^b^	26.07 ± 1.99^c^
21 d	26.06 ± 1.12^b^	41.09 ± 0.67^b^
35 d	29.82 ± 1.50^a^	46.07 ± 2.86^b^
60 d	30.36 ± 1.61^a^	52.88 ± 3.72^a^

Data without the same superscripts (a–d) differ significantly (*P* < 0.05).

## Data Availability

The data used to support the findings of this study are available from the corresponding author upon request.
